# Modern and Ancestral Genotypes of *Mycobacterium tuberculosis* from Andhra Pradesh, India

**DOI:** 10.1371/journal.pone.0027584

**Published:** 2011-11-17

**Authors:** Shirly K. Thomas, Chitra C. Iravatham, Bottu Heleena Moni, Ashutosh Kumar, Bandaru V. Archana, Mohammad Majid, Yerra Priyadarshini, Pittu Sandhya Rani, Vijayalakshmi Valluri, Seyed E. Hasnain, Niyaz Ahmed

**Affiliations:** 1 Institute of Life Sciences, University of Hyderabad Campus, Hyderabad, India; 2 Bhagwan Mahavir Hospital and Research Centre, AC Guards, Hyderabad, India; 3 Pathogen Biology Laboratory, Department of Biotechnology, School of Life Sciences, University of Hyderabad, Hyderabad, India; 4 Blue Peter Research Centre – Lepra India, Cherlapally, Hyderabad, India; 5 School of Biological Sciences, Indian Institute of Technology, New Delhi, India; 6 Institute of Biological Sciences, University of Malaya, Kuala Lumpur, Malaysia; Charité-University Medicine Berlin, Germany

## Abstract

Traditionally, the distribution of the *Mycobacterium tuberculosis* genotypes in India has been characterized by widespread prevalence of ancestral lineages (TbD1+ strains and variants) in the south and the modern forms (TbD1^−^ CAS and variants) predominating in the north of India. The pattern was, however, not clearly known in the south-central region such as Hyderabad and the rest of the state of Andhra Pradesh where the prevalence of both tuberculosis (TB) and human immunodeficiency virus (HIV) infection is one of the highest in the country; this area has been the hotspot of TB vaccine trials. Spoligotyping of 101 clinical isolates obtained from Hyderabad and rural Andhra Pradesh confirmed the occurrence of major genogroups such as the ancestral (or the TbD1+ type or the East African Indian (EAI) type), the Central Asian (CAS) or Delhi type and the Beijing lineage in Andhra Pradesh. Sixty five different spoligotype patterns were observed for the isolates included in this study; these were further analyzed based on specific genetic signatures/mutations. It was found that the major genogroups, CAS and “ancestral,” were almost equally prevalent in our collection but followed a north-south compartmentalization as was also reported previously. However, we observed a significant presence of MANU lineage in south Andhra Pradesh, which was earlier reported to be overwhelmingly present in Mumbai. This study portrays genotypic diversity of *M. tuberculosis* from the Indian state of Andhra Pradesh and provides a much needed snapshot of the strain diversity that will be helpful in devising effective TB control programs in this part of the world.

## Introduction

Tuberculosis (TB) today constitutes the second major cause of death due to infectious diseases. India being the hotspot region for TB witnesses one of the highest incidence rates although the mortality figures are on the decline due to effective implementation of the control programs. Nevertheless, the scientific challenge in TB control has become complicated with the emergence of new frightening forms of tuberculosis – the extensively drug resistant tuberculosis (XDR-TB) and the HIV-TB co-infection.

Correct identification of the underlying strains is of paramount importance in devising TB control strategies. Spoligotyping is one of the potentially powerful tools for simultaneous detection and differentiation of *M. tuberculosis* complex lineages [1]. Studies using multiple markers revealed predominance of ancestral (TbD1+) and modern (TbD1−) genogroups of *M. tuberculosis* strains in India [2]. The signature deletions RD239 and RD 750 [3] are specifically useful to define isolates belonging to the ‘Indo-Oceanic’ (TbD1+) [3] or ‘East African/Indian’ – EAI [2] and CAS lineages. Genotyping studies from India revealed a C→T specific silent mutation in 65^th^ codon of the *pncA* gene which is specific for the CAS (Central Asian) lineage [4]. A new, ancient clade of strains, called as ‘MANU’ was identified in India which belongs to the ancestral family of principle genetic group (PGG) - 1 [5], [6] and is heavily concentrated in Mumbai [7]. It is tentatively subdivided in to MANU1 (ST100; loss of spacer 34), MANU2 (ST 54; loss of spacers 33, 34) and MANU3 (ST1378; deletion of spacers 34–36) [5], [7], [8], [9]. It has been suggested that spoligotypes evolve through the successive loss of spacer DNA sequences. In addition to the loss of spacer 34, MANU [this study] and ‘ancestral’ EAI or Indo-oceanic lineages [3] are characterized by the deletion of RD 239 and an intact TbD1 region; it thus appears that MANU and EAI lineages are closely related or were derived from a last common ancestor.

Andhra Pradesh, with a population of about 80 million is one of India's states with high prevalence of HIV/AIDS. Patients with latent TB infection are at higher risk of progression if they are co-infected with HIV. A few TB vaccine trials have been initiated in this region although the repertoire of circulating strains is largely unknown. Thus, the aim of this study was to characterize the predominant genotypes responsible for TB in urban and rural Andhra Pradesh and to generate a preliminary, baseline data for further epidemiological and infection control studies.

## Materials and Methods

### Ethics statement

All the mycobacterial strains analyzed in this study were available as a part of routine TB testing and surveillance programs being implemented at different centres and, therefore, human ethics committee (Institutional Review Board equivalent in India) approvals were not mandatory (also since no human biological samples were collected here). The study was part of a long term open ended project originally approved by the Institutional Biosafety Committee (IBSC) of the School of Life Sciences, University of Hyderabad.

### Bacterial isolates

A total of 101 clinical isolates of *M. tuberculosis* representing TB patients from urban and rural Andhra Pradesh were analyzed in this study; 59 of these originated from a collection of MDR isolates cultured during 2000–2005 as a part of Hyderabad Urban DOTS program. The other isolates represented randomly selected collection at microscopy centres operating under the aegis of the revised national tuberculosis control program (RNTCP) (Table 1). Smear positive sputum specimens received for routine diagnosis were processed by modified Petroff's method and cultured on Lowenstein–Jensen slants at 37°C for 6–8 weeks. Drug susceptibility testing was performed by the absolute concentration (MIC) method for the anti-TB drugs, namely, isoniazid [H], ethambutol [E], rifampicin [R] and by resistance ratio method (RR method) for other drugs such as streptomycin [S], according to the protocol of Tuberculosis Research Centre, Chennai, India (TRC) [10]. Multi Drug Resistance (MDR) phenotype was defined as resistance to both isoniazid and rifampicin.

**Table 1:** Details of the *M. tuberculosis* isolates studied herein.

Isolate/batch identity (Numbers)	Geographical origin	Selection criteria	Source centre type	Important genogroups
AP01 to AP59 (59)	Hyderabad and Ranga Reddy district (urban group)	Pulmonary TBMDR isolates	Hyderabad urban DOTS	CASEAIBeijing
AP60 to AP101 (42)	Chittoor district (rural group)	Pulmonary TBRandom selection/cross sectional(No resistotyping data)	RNTCP rural microscopy centres	EAIMANUCAS

### DNA isolation and spoligotyping

**Figure 1:**
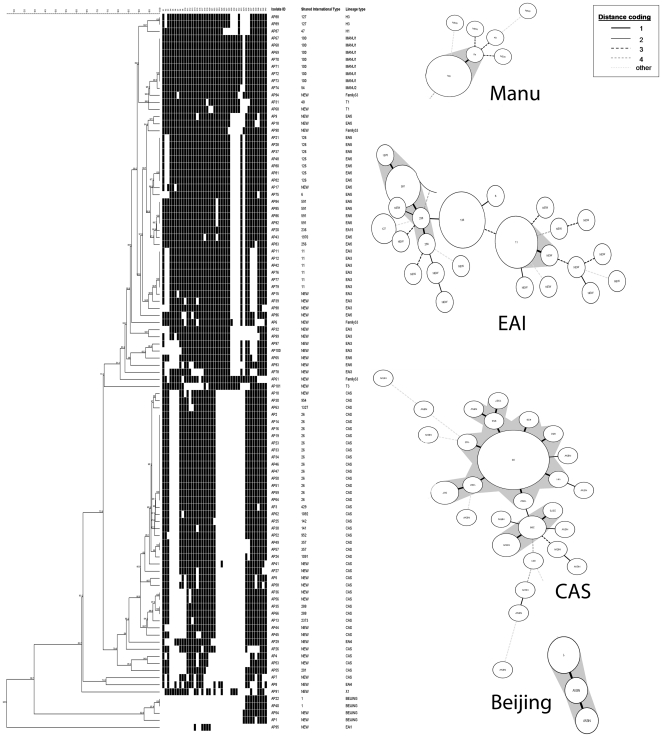
Genetic affinities within the *M. tuberculosis* isolates based on spoligotyping. Different clades corresponding to prevalent genotypes are prominently highlighted. In the inset is the distance coding convention relevant to the genetic relatedness of different isolates within a clade.

Isolation of DNA was carried out as previously described [11]. Spoligotyping was performed according to the standard method [1] and with the help of commercially available line probe arrays (Isogen Biosciences BV, Maarssen, the Netherlands). The hybridization pattern was visualized after incubation with streptavidin peroxidase using an enhanced chemiluminescence detection system (Amersham) followed by exposure to an X-ray film (Hyperfilm ECL, Amersham). Results were expressed as presence (n) or absence (0) of each of the 43 spacers and converted to an octal code format according to Dale *et al* [12]. The data were then compared to the SpolDB4 or SITVIT database and ‘Spotclust’ [12], [13], [14] to assign strain families based on standard definition/convention [13], [14]. The isolates with spoligotype patterns present in the SITVIT database (SpolDB4) were automatically labeled with a ‘shared type’ number and unique profiles were mentioned as ‘new’. The spoligotyping results were further analyzed with the help of Bionumerics® software program (Applied Maths, Belgium); a dendrogram was constructed (Figure 1) by un-weighted pair group method using arithmetic averages (UPGMA) [15].

### TbD1 analysis

The presence (TbD1+) or absence (TbD1−) of the TbD1 region was analyzed by PCR using two primer sets complementary to the sequence of the deleted region or complementary to the internal sequence of the intact TbD1 region [16]. TbD1+ isolates generated a PCR product primed by the internal primers (2,153 bp), whereas the TbD1− isolates generated a PCR product based on the flanking primers (500 bp).

### RD 239 and RD 750 deletion analysis

The RD 239 and RD 750 specific primers were used to amplify specific signature DNA and assign strains to either the EAI lineage or the CAS lineage [3], [17]. RD239 deletions were denoted by an amplicon of size 888 bp and intact regions were scored based on the presence of an amplicon of 1730 bp. Similarly, an RD750 specific deletion resulted in a 734 bp PCR product and an intact region generated a product of 1533 bp.

### PCR-RFLP and Sequencing

**Figure 2:**
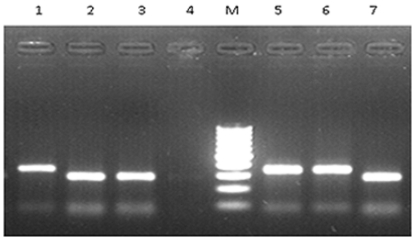
PCR-RFLP analysis of the *pncA* gene after restriction digestion with *Bse*LI. The *pncA* gene was analyzed for a silent mutation in the 65^th^ codon at the 195th bp. Mutated alleles correspond to lanes 1, 5 and 6 (344 bp product and one minor fragment of 81 bp). Lanes 2, 3 and 7 denote wild type pattern (280 bp product and two minor fragments of 81 and 64 bp sizes) while lane 4 represents a negative control. Lane M corresponds to the profiling of a 100 bp DNA molecular weight marker.

The *pncA* gene was analyzed for a silent mutation in 65^th^ codon at the 195^th^ bp [4]. Restriction digestion using BseLI enzyme (Fermentas) differentiated the mutated and wild type alleles. Gel electrophoresis was performed to resolve the digested products in 1.5% agarose gel (Figure 2).

## Results and Discussion

Spoligotyping of 101 clinical *M. tuberculosis* isolates revealed 65 distinct spoligopatterns. Thirty nine out of 101 clinical isolates were represented by a unique pattern whereas sixty two isolates segregated into 26 different clusters or branches (Figure 1). Three clusters comprising of 13 isolates, 7 isolates and 7 isolates belonged to ST 26, ST126 and ST 100 respectively, followed by 6 isolates belonging to ST11 and 4 isolates belonging to ST591. Two clusters (ST1, ST288) were formed with only 2 isolates each. Two other clusters (ST127 and ST357) also comprised only 2 isolates each and seventeen spoligopatterns represented single isolates (Figure 1). Sixty three percent of all clusters/branches had only one isolate and ∼39% of all the isolates were not previously reported in the SpolDB4, SITVIT or ‘Spotclust’ and were identified as ‘new’. These unique patterns were variously affiliated to CAS, EAI1, EAI3, EAI4, EAI5, family33, X1, T1, T3 and Beijing genogroups.

The predominant spoligo families corresponded to CAS (∼40%), EAI (∼38%) and MANU (∼8%). The CAS and EAI groups together comprised 77.22% of the total isolates. Other families represented were Beijing (∼4%), family33 (4%), Haarlem (H) [∼3%], X (1%) and Tuscany (T) [∼3%]. The EAI family comprised of 55.26% EAI5, 36.84% EAI3, 5.26% EAI4, and ∼2.63% EAI1. While the ST26 belonged to CAS1_Delhi lineage, ST11 was grouped under EAI3_IND lineage. ST100 belonged to MANU clade and ST126 was grouped under EAI5 (Figure 1)

The drug susceptibility testing of the 32 representative isolates obtained by random selection out of the urban MDR group (Table 1) showed 25 isolates (78.12%) of the families CAS, Beijing, EAI5, EAI3 and EAI4, were MDR. Although a single isolate from EAI4 was found to be resistant to streptomycin and isoniazid (but sensitive to ethambutol and rifampicin), 6 other isolates belonging to different genotypes were found sensitive.

Random samples representing the EAI/Indo-oceanic lineage were reconfirmed based on 1) the presence of RD239, 2) an intact RD750 region, 3) an intact TbD1 region together with 4) non-mutated codon 65 of the *pncA* gene. Similarly, representative isolates from the CAS lineage were reconfirmed by 1) an intact RD239 region, 2) a deleted RD750 region, 3) the TbD1deletion and 4) mutations in the codon 65 of the *pncA* gene. The seven isolates belonging to MANU1 lineage were analyzed for mutations in the *pncA* gene. PCR-RFLP for the *pncA* gene (Figure 2) was developed and standardized in house. Six of the MANU1 isolates had a wild type gene and a single isolate was found to be mutated at codon 65. Sequencing of the amplicons from four representative MANU1 isolates confirmed the PCR-RFLP data (Gen bank Accessions for the *pnc*A: GU817406, GU817407, GU817408, GU817409).

Spoligotype data from this study indicate that the known lineages ST26, ST100, ST126 and ST11 predominate in Andhra Pradesh; this trend was also reported from elsewhere [2], [6], although the most significant finding from our data was the occurrence of orphan spoligotypes (new) not identified in such high proportion in any previous studies from India.

According to other studies from India, ST26, ST11, ST1 and ST126 together accounted for a major proportion of all the isolates studied from this country [2], [4], [5], [6], [7], [8]. Although, traditionally, ST100 was found to be a minor clade in different studies from India, except its high occurrence in Mumbai [7], we in our study found that the ancestral isolates of ST126 and ST100 type (MANU) could be much more concentrated in southern Andhra Pradesh (Chittoor district). Moreover, the most predominant spoliogotype, ST26 (CAS1_Delhi), was found in 12.87% of all the clustered isolates and was highly prevalent in Hyderabad region. Shared type ST11 which belongs to EIA3_IND clade accounted for only 6% of all the clustered isolates.

For this collection, the CAS (TbD1−) and EAI (TbD1+) lineages were found almost in an equal ratio (∼40% CAS and ∼38% EAI). However, if we take in to account the MANU types, then the incidence of ancestral type isolates (TbD1+) becomes clearly dominant. It has previously been shown that the CAS lineage predominates in north and EAI in south India [2], [4], [5], [6]. In our earlier study carried out on a nation wide sample, EAI genotypes were clearly less predominant in north India (32%), followed by 52% in central India and 80% in south India [2]. The present data are in agreement with such findings and also with respect to the prevalence of Beijing strains [18] that accounted to be only about 3–5% [2]. We did not find any Beijing isolates in our rural group and it is possible that southern Andhra Pradesh may not have any prevalence of this exotic genotype.

Both mutated and wild type forms of the *pncA* gene were present in the MANU types. Thus, the CAS - lineage specific silent (C→T) mutation [4] was also noticed in the MANU isolates characterized in this study. Since both the EAI and MANU lineages have RD239 deleted along with an intact TbD1, it is interesting to know whether EAI or Indo-oceanic lineage is indeed evolved from MANU1 through successive loss of spacer DNA sequences. Since our data are based on limited isolates, there is a need for an elaborate study to understand this possibility as well as the significance of MANU strains with mutated *pncA* gene. As described above, the codon 65 mutation seems to be common among ST26 and a single ST100 genotype, however, it is difficult to relate them based solely on single gene mutations. On the other hand, TbD1 positivity and deletion of RD239 constitute much stronger evidence to portray them as ancestrally very closely related.

Both the modern and ancestral *M. tuberculosis* strains are prevailing in this region with a north-south compartmentalization, respectively, and the isolates show a high degree of spoligotype signature diversity. The present data once again remind of the possibility that the ancestral strains are somehow more adapted to southern peninsula. Although we did not analyze our samples based on demographic data of patients, it is possible that the cosmopolitan nature of Hyderabad population could have lead to more diverse spoligotype patterns and representation of different lineages without clear dominance of a single lineage. Although ‘modern’ (TbD1−) *M. tuberculosis* strains are far more prevalent worldwide, the ancestral clones of *M. tuberculosis* are responsible for a majority of TB cases in India with the exception of major metropolitan cities. Our results therefore corroborate with the findings of Gutierrez *et al.* [2] who reported similar trend on the basis of genotyping of a ‘national collection’ of isolates.

To sum up, this study has proven useful in identifying the predominant spoligotypes responsible for disease transmission in Andhra Pradesh. Significant presence of the ancestral type bacteria in the TB patients from Andhra Pradesh as shown here assumes importance in the light of our earlier espousal [19], [20], [21] and one recent finding that *M. tuberculosis* belonging to the ancestral lineage (EAI) could show reduced transmission as compared to other lineages [22]. This perhaps explains why the Indian population has never suffered with institutionalized TB outbreaks as seen in some other parts of the world where ancestral type bacteria are not so prevalent. Having said this, we sincerely wish our observations may form path forward to more rigorous future studies, based on whole genome sequencing of the underlying isolates, to obtain better picture of the existing diversity, transmission patterns and the preponderance of drug resistant strains in this high-incidence region.
